# Mass spectrometry based on a coupled Cooper-pair box and nanomechanical resonator system

**DOI:** 10.1186/1556-276X-6-570

**Published:** 2011-10-31

**Authors:** Cheng Jiang, Bin Chen, Jin-Jin Li, Ka-Di Zhu

**Affiliations:** 1 Key Laboratory of Artificial Structures and Quantum Control (MOE), Department of Physics, Shanghai Jiao Tong University, 800 Dong Chuan Road, Shanghai 200240, China

## Abstract

Nanomechanical resonators (NRs) with very high frequency have a great potential for mass sensing with unprecedented sensitivity. In this study, we propose a scheme for mass sensing based on the NR capacitively coupled to a Cooper-pair box (CPB) driven by two microwave currents. The accreted mass landing on the resonator can be measured conveniently by tracking the resonance frequency shifts because of mass changes in the signal absorption spectrum. We demonstrate that frequency shifts induced by adsorption of ten 1587 bp DNA molecules can be well resolved in the absorption spectrum. Integration with the CPB enables capacitive readout of the mechanical resonance directly on the chip.

## 1 Introduction

Nanoelectromechanical systems (NEMS) offer new prospects for a variety of important applications ranging from semiconductor-based technology to fundamental science [1]. In particular, the minuscule masses of NEMS resonators, combined with their high frequencies and high resonance quality factors, are very appealing for mass sensing [2-7]. These NEMS-based mass sensing employs tracking the resonance frequency shifts of the resonators due to mass changes. The most frequently used techniques for measuring the resonance frequency are based on optical detection [8]. Though inherently simple and highly sensitive, this technique is susceptible to temperature fluctuation noise because it usually generates heat and heat conduction. On the other hand, it has experimentally been demonstrated that capacitive detection is less affected to noise than optical detection in ambient atmosphere [9]. Capacitive detection is realized by connecting NEMS resonator with standard microelectronics, such as complementary metal-oxide-semiconductor (CMOS) circuitry [10]. Here, we propose a scheme for mass sensing based on a coupled nanomechanical resonator (NR)-Cooper-pair box (CPB) system.

The basic superconducting CPB consists of a low-capacitance superconducting electrode weakly linked to a superconducting reservoir by a Josephson tunnel junction. Owing to its controllability [11-14], a CPB has been proposed to couple to the NR to drive an NR into a superposition of spatially separated states and probe the decay of the NR [15], to prepare the NR in a Fock state and perform a quantum non-demolition measurement of the Fock state [16], and to cool the NR to its ground state [17]. Recently, this coupled CPB-NR system has been realized in experiments [18,19] and the resonance frequency shifts of the NR could be monitored by performing microwave (MW) spectroscopy measurement. Based on the above-mentioned achievements, in this article, we investigate the signal absorption spectrum of the CPB qubit capacitively coupled to an NR in the simultaneous presence of a strong control MW current and a weak signal MW current. Theoretical analysis shows that two sideband peaks appear at the signal absorption spectrum, which exactly correspond to the resonance frequency of the NR. Therefore, the accreted mass landing on the NR can be weighed precisely by measuring the frequency shifts because of mass changes of the NR in the signal absorption spectrum. Similar mass sensing scheme has been proposed recently in a hybrid nanocrystal coupled to an NR by our group [20], which is based on a theoretical model. However, recent experimental achievements in the coupled CPB-NR system [18,19] make it possible for our proposed mass sensing scheme here to be realized in future.

## 2 Model and theory

**Figure 1:**
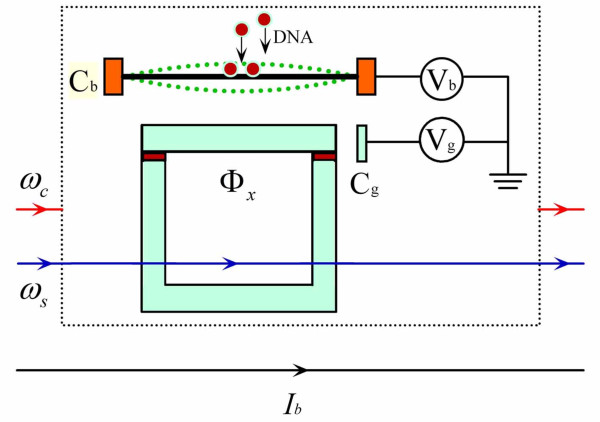
**Schematic diagram of an NR capacitively coupled to a CPB**. Two MW currents with frequency *ω*_c _and *ω*_s _and a direct current *I*_b _are applied in the MW line to control the magnetic flux *Φ_x _*through the CPB loop.

In our CPB-NR composite system shown schematically in Figure 1, the NR is capacitively coupled to a CPB qubit consisting of two Josephson junctions which form a SQUID loop. A strong control MW current and a weak signal MW current are simultaneously applied in a MW line through the CPB to induce the oscillating magnetic fields in the Josephson junction SQUID loop of the CPB qubit. Besides, a direct current *I_b _*is also applied to the MW line to control the magnetic flux through the SQUID loop and thus the effective Josephson coupling of the CPB qubit. The Hamiltonian of our coupled CPB-NR system reads:

(1) $$H=H_{\text{CPB}}+H_{\text{NR}}+H_{\text{int}},$$

(2) $$H_{\text{CPB}}=\frac{1}{2}\hbar \omega_{\text{q}}\sigma_{z}-\frac{1}{2}E_{\text{J0}}\cos\left[\frac{\pi \Phi_{x}\left(t\right)}{\Phi_{0}}\right]\sigma_{x},$$

(3) $$H_{\text{NR}}=\hbar \omega_{n}a^{†}a,$$

(4) $$H_{\text{int}}=\hbar \lambda \left(a^{†}+a\right)\sigma_{z}.$$

where *H*_CPB _is the Hamiltonian of the CPB qubit described by the pseudospin -1/2 operators *σ_z _*and *σ_x _*= *σ*_+ _+ *σ*_-_. *ω*_q _= 4*E*_c_(2*n*_g _- 1)/*ħ *is the electrostatic energy difference and *E*_J0 _is the maximum Josephson energy. Here, *E*_C _= *e*^2^/2*C_Σ _*is the charging energy with *C_Σ _*= *C*_b _+ *C*_g _+ 2*C*_J _being the CPB island's total capacitance and *n_g _*= (*C*_b_*V*_b _+ *C*_g_*V*_g_)/(2*e*) is the dimensionless polarization charge (in units of Cooper pairs), where *C*_b _and *V*_b _are, respectively, the capacitance and voltage between the NR and the CPB island, *C*_g _and *V*_g _are, respectively, the gate capacitance and voltage of the CPB qubit, and *C*_J _is the capacitance of each Josephson junction. Displacement (by *x*) of the NR leads to linear modulation of the capacitance between NR and CPB, *C*_b_(*x*) ≈ *C*_b_(0) + (∂*C*_b_/∂*x*)*x*, which modulates the electrostatic energy of the CPB qubit, resulting in the capacitive coupling constant $\lambda =\frac{4n_{\text{g}}^{\text{NR}}E_{\text{C}}}{\hbar }\frac{1}{C_{\text{b}}}\frac{\partial C_{\text{b}}}{\partial x}\Delta x_{zp}$, where $n_{\text{g}}^{\text{NR}}=C_{\text{b}}V_{\text{b}}∕2e$ and $\Delta x_{zp}=\sqrt{\hbar ∕2m\omega_{n}}$ is the zero-point uncertainty of the NR with effective mass *m *and resonance frequency *ω_n_*. The coupling between the MW line and the CPB qubit in the second term of Equation 2 results from the totally externally applied magnetic flux *Φ_x_*(*t*) = *Φ*_q_(*t*) + *Φ*_b _through the CPB qubit loop of an effective area *S *with *Φ*_0 _= *h*/(2*e*) being the flux quantum. Here, *Φ*_q_(*t*) = *μ*_0_*SI*(*t*)/(2*πr*), with *r *being the distance between the MW line and the qubit and *μ*_0 _being the vacuum permeability. *Φ*_q_(*t*) and *Φ*_b _are controlled, respectively, by the MW current $I\left(t\right)={ℰ}_{\text{c}}\text{cos}\left(\omega_{\text{c}}t\right)+{ℰ}_{\text{s}}\text{cos}\left(\omega_{\text{s}}t+\delta^{\prime }\right)$ and the direct current *I*_b _in the MW line. For convenience, we assume the phase factor *δ' *= 0 because it is not difficult to demonstrate that the results of this article are not dependent on the value of *δ'*. By adjusting the direct current *I*_b _and the MW current *I*(*t*) such that *Φ*_b _≫ *Φ*_q _(*t*) and *πΦ*_b_/*Φ*_0 _= *π */2, we can obtain $E_{\text{J}}\text{cos}\left[\frac{\pi \Phi_{x}\left(t\right)}{\Phi_{0}}\right]\approx -E_{\text{J}}\frac{\pi \Phi_{\text{q}}\left(t\right)}{\Phi_{0}}.$ In a rotating frame at the control frequency *ω*_c_, the total Hamiltonian can now be written as

(5) $$\begin{array}{c} H=\frac{1}{2}\hbar \Delta \sigma_{z}+\hbar \omega_{n}a^{†}a+\hbar \lambda \left(a^{†}+a\right)\sigma_{z}+\hbar \Omega \left(\sigma_{+}+\sigma_{-}\right)\\ \;\;+\mu {ℰ}_{s}\left(\sigma_{+}e^{-i\delta t}+\sigma_{-}e^{i\delta t}\right), \end{array}$$

where *Δ *= *ω*_q _- *ω*_c _is the detuning of the qubit resonance frequency and the control current frequency, *δ *= *ω*_s _- *ω*_c _is the detuning of the signal current and the control current, *μ *= *μ*_0_*SE*_J0_/(8*rΦ*_0_) is the effective 'electric dipole moment' of the qubit, and $\Omega =\mu {ℰ}_{\text{c}}∕\hbar$ is the effective 'Rabi frequency' of the control current.

The dynamics of the coupled CPB-NR system in the presence of dissipation and dephasing is described by the following master equation [21]

(6) $$\frac{d\rho }{dt}=-\frac{i}{\hbar }\left[H,\rho \right]+\frac{1}{2T_{1}}ℒ\left[\sigma_{-}\right]+\frac{\gamma }{2}ℒ\left[a\right]+\frac{1}{4\tau_{\phi }}ℒ\left[\sigma_{z}\right],$$

where *ρ *is the density matrix of the coupled system, *T*_1 _is the qubit relaxation time, *τ_ϕ _*is the qubit pure dephasing time, and *γ *is the decay rate of the NR which is given by *γ *= *ω_n_*/*Q*. ℒ[D], describing the incoherent decays, is the Lindblad operator for an operator and is given by:

(7) $$ℒ\left[D\right]=2D\rho D^{†}-D^{†}D\rho -\rho D^{†}D.$$

Using the identity $\langle \dot{O}\rangle =Tr(O\dot{\rho })$ for an operator *O *and a density matrix *ρ *in Equation 6, we obtain the following Bloch equations for the coupled CPB-NR system:

(8) $$\begin{array}{c} \frac{d\left\langle \sigma_{-}\right\rangle }{dt}=-\left(\frac{1}{T_{2}}+i\Delta \right)\left\langle \sigma_{-}\right\rangle -i\left\langle Q\sigma_{-}\right\rangle +i\Omega \left\langle \sigma_{z}\right\rangle \\ \;\;\;\;+\frac{i}{\hbar }\mu {ℰ}_{s}\left\langle \sigma_{z}\right\rangle e^{-i\delta t}, \end{array}$$

(9) $$\begin{array}{c} \frac{d\left\langle \sigma_{z}\right\rangle }{dt}=-\frac{1}{T_{1}}\left(\left\langle \sigma_{z}\right\rangle +1\right)-2i\Omega \left(\left\langle \sigma_{+}\right\rangle -\left\langle \sigma_{-}\right\rangle \right)\\ \;\;\;\;-2\frac{i}{\hbar }\mu \left({ℰ}_{s}\left\langle \sigma_{+}\right\rangle e^{-i\delta t}-{ℰ}_{s}^{*}\left\langle \sigma_{-}\right\rangle e^{i\delta t}\right), \end{array}$$

(10) $$\frac{d^{2}\left\langle Q\right\rangle }{dt^{2}}+\gamma \frac{d\left\langle Q\right\rangle }{dt}+\omega_{r}^{2}\left\langle Q\right\rangle =-4\omega_{r}^{3}\lambda_{0}\left\langle \sigma_{z}\right\rangle ,$$

where $\lambda_{0}=\frac{\lambda^{2}}{\omega_{n}^{2}}$ and *T*_2 _is the qubit dephasing time satisfying

(11) $$\frac{1}{T_{2}}=\frac{1}{2T_{1}}+\frac{1}{\tau_{\phi }}.$$

Note that if the pure dephasing rate is neglected, i.e., $\frac{1}{\tau_{\phi }}=0$, then *T*_2 _= 2*T*_1_. In order to solve the above equations, we first take the semiclassical approach by factorizing the NR and CPB qubit degrees of freedom, i.e., 〈*Qσ*_-_〉 = 〈*Q*〉 〈*σ*_-_〉, which ignores any entanglement between these systems. For simplicity, we define *p *= *μσ*_-_, *k *= *σ_z _*and then we have

(12) $$\frac{dp}{dt}=\left[-\frac{1}{T_{2}}-i\left(\Delta +\left\langle Q\right\rangle \right)\right]p+i\frac{\mu^{2}kℰ}{\hbar },$$

(13) $$\frac{dk}{dt}=-\frac{1}{T_{1}}\left(k+1\right)-\frac{4}{\hbar }\text{Im}\left(p{ℰ}^{*}\right),$$

(14) $$\frac{d^{2}\left\langle Q\right\rangle }{dt^{2}}+\gamma \frac{d\left\langle Q\right\rangle }{dt}+\omega_{r}^{2}\left\langle Q\right\rangle =-4\lambda_{0}\omega_{r}^{3}k$$

where $ℰ={ℰ}_{c}+{ℰ}_{s}e^{-i\delta t}$. In order to solve the above equations, we make the ansatz 〈*p*(*t*)〉 = *p*_0 _+ *p*_1_*e*^-*iδt *^+ *p*_-1_*e^iδt^*, 〈*k*(*t*)〉 = *k*_0 _+ *k*_1_*e*^-*iδt *^+ *k*_-1_*e^iδt^*, and 〈*Q*(*t*)〉 = *Q*_0 _+ *Q*_1_*e*^-*iδt *^+ *Q*_-1_*e^iδt ^*[22]. Upon substituting these equations into Equations 12-14 and upon working to the lowest order in ${ℰ}_{s}$ but to all orders in ${ℰ}_{c}$, we obtain in the steady state:

(15) $$p_{1}=\frac{\mu^{2}{ℰ}_{s}T_{2}k_{0}}{\hbar }\frac{2T_{1}∕T_{2}B\left(\delta_{0}+2i\right)\left(C+\Omega_{c}^{2}\right)+E\left(B-\delta_{0}\right)}{AE\left(B-\delta_{0}\right)}.$$

where

(16) $$\begin{array}{ll} A & =\Delta_{c}-4\lambda_{0}\omega_{0}k_{0}-\delta_{0}-i,\;\\ B & =\Delta_{c}-4\lambda_{0}\omega_{0}k_{0}+\delta_{0}+i,\;\\ C & =4\lambda_{0}\omega_{0}k_{0}\eta \Omega_{c}^{2}∕\left(\Delta_{c}-4\lambda_{0}\omega_{0}k_{0}-i\right),\;\\ D & =4\lambda_{0}\omega_{0}k_{0}\eta \Omega_{c}^{2}∕\left(\Delta_{c}-4\lambda_{0}\omega_{0}k_{0}+i\right),\;\\ E & =2T_{1}∕T_{2}A\left(D+\Omega_{c}^{2}\right)-2T_{1}∕T_{2}B\left(C+\Omega_{c}^{2}\right)\;\\ & -AB\left(T_{1}∕T_{2}\delta_{0}+i\right).\;\\ & \; \end{array}$$

Here, dimensionless variables *ω*_0 _= *ω_r_T*_2_, *γ*_0 _= *γT*_2_, *Ω_c _*= *ΔT*_2_, and *Δ_c _*= *ΔT*_2 _are introduced for convenience and the auxiliary function

(17) $$\eta =\frac{\omega_{0}^{2}}{\omega_{0}^{2}-i\gamma_{0}\delta_{0}-\delta_{0}^{2}}.$$

The population inversion *k*_0 _of the CPB is determined by

(18) $$\left(k_{0}+1\right)[{(\Delta_{c}-4\lambda_{0}\omega_{0}k_{0})}^{2}+1]+4\Omega_{c}^{2}k_{0}\frac{T_{1}}{T_{2}}=0.$$

*p*_1 _is a parameter corresponding to the linear susceptibility $\chi^{\left(1\right)}\left(\omega_{s}\right)=p_{1}∕{ℰ}_{s}=\left(\mu^{2}T_{2}∕\hbar \right)\chi \left(\omega_{s}\right)$, where the dimensionless linear susceptibility *χ*(*ω_s_*) is given by

(19) $$\chi \left(\omega_{s}\right)=\frac{2T_{1}∕T_{2}B\left(\delta_{0}+2i\right)\left(C+\Omega_{c}^{2}\right)+E\left(B-\delta_{0}\right)}{AE\left(B-\delta_{0}\right)}k_{0}.$$

The real and imaginary parts of *χ*(*ω_s_*) characterize, respectively, the dispersive and absorptive properties.

The coupled CPB-NR system has been proposed to measure the vibration frequency of the NR by calculating the absorption spectrum [23]. On the other hand, NRs have widely been used as mass sensors by measuring the resonant frequency shift because of the added mass of the bound particles. The mass sensing principle is simple. NRs can be described by harmonic oscillators with an effective mass *m*_eff_, a spring constant *k*, and a mechanical resonance frequency $\omega_{n}=\sqrt{k∕m_{\text{eff}}}$. When a particle adsorbs to the resonator and significantly increases the resonator's effective mass, therefore, the mechanical resonance frequency reduces. Mass sensing is based on monitoring the frequency shift *Δω *of *ω_n _*induced by the adsorption to the resonator. The relationship between *Δω *with the deposited mass *Δm *is given by

(20) $$\Delta m=-\frac{2m_{\text{eff}}}{\omega_{n}}\Delta \omega ={ℛ}^{-1}\Delta \omega ,$$

where $ℛ={\left(-2m_{\text{eff}}∕\omega_{n}\right)}^{-1}$ is defined as the mass responsivity. However, the measurement techniques are rather challenging. For example, electrical measurement is unsuitable for mass detections based on very high frequency NRs because of the generated heat effect [24]. For optical detection, as device dimensions are scaled far below the detection wavelength, diffraction effects become pronounced and will limit the sensitivity of this approach [25]. Moreover, in any actual implementation, frequency stability of the measuring system as well as various noise sources, including thermomechanical noise generated by the internal loss mechanisms in the resonator and Nyquist-Johnson noise from the readout circuitry [3,26] will also impose limits to the sensitivity of measurement. Here, we can determine the frequency shifts with high precision by the MW spectroscopy measurement based on our coupled CPB-NR system.

## 3 Numerical results and discussion

In what follows, we choose the realistically reasonable parameters to demonstrate the validity of mass sensing based on the coupled CPB-NR system. Typical parameters of the CPB (charge qubit) are *E*_C_/*ħ *= 40 GHz and *E*_J0_/*ħ *= 4 GHz such that *E*_C _≫ *E*_J _[27]. Experiments by many researchers have demonstrated CPB eigenstates with excited state lifetime of up to 2 *μ*s and coherence times of a superpositions states as long as 0.5 *μ*s, i.e., *T*_1 _= 2*μ*s, and *T*_2 _= 0.5 *μ*s [13,28,29]. NR with resonance frequency *ω_n _*= 2*π *× 133 MHz, quality factor *Q *= 5000, and effective mass *m*_eff _= 73 fg has been used for zeptogram-scale mass sensing [5]. Besides, coupling constant *λ *between the CPB and NR can be chosen as *λ *= 0.1*ω_n _*= 2*π *× 13.3 MHz [16]. We assume *S *= 1 *μ*m^2^, *r *= 10 *μ*m, and ${ℰ}_{\text{c}}=200\mu \text{A}$[30], therefore, we can obtain *μ*/*ħ *= *μ*_0_*SE*_J0_/(8*ħrϕ*_0_) ≈ 30 GHzA^-1 ^and $\Omega_{\text{c}}=\Omega T_{2}=\left(\mu ∕\hbar \right){ℰ}_{\text{c}}T_{2}=3$. The experiments of our proposed mass sensing scheme should be done *in situ *within a cryogenically cooled, ultrahigh vacuum apparatus with base pressure below 10^-10 ^Torr.

**Figure 2:**
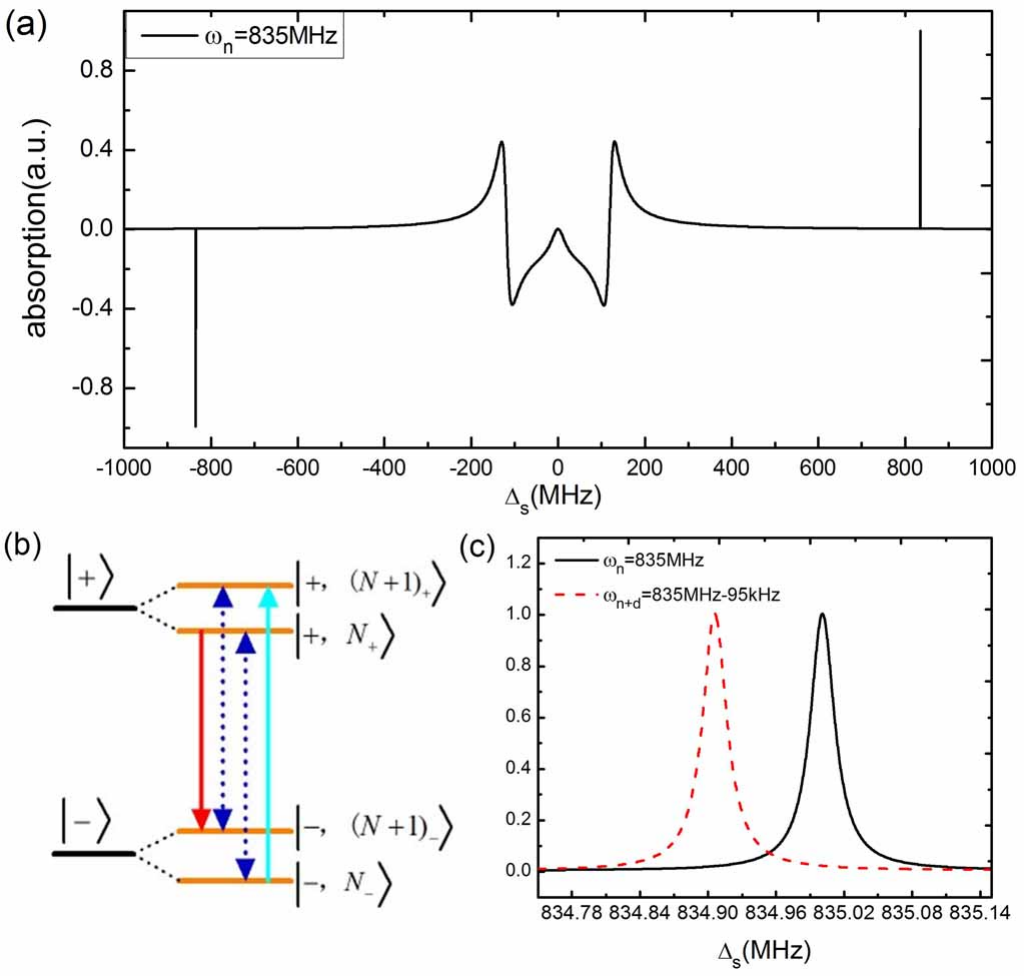
**Scaled absorption spectrum of the signal current as a function of the detuning ***Δ*_s _**and energy level diagram of the coupled system**. **(a) **Scaled absorption spectrum of the signal current as a function of the detuning *Δ*_s _without landing any masses on the NR. **(b) **The energy level diagram of the CPB coupled to an NR. **(c) **Signal absorption spectrum as a function of *Δ*_s _before (black solid line) and after (red dashed line) a binding event of ~ 10 functionalized 1587 bp long dsDNA molecules. Frequency shift of 95 kHz can be well resolved in the spectrum. Other parameters used are *ω_n _*= 835 MHz, *λ*_0 _= 0.01, *Δ*_c _= 0, *Q *= 5000, *T*_1 _= 0.25 *μ*s, *T*_2 _= 0.05 *μ*s, and *Ω_c _*= 3.

Firstly, we would show the principle of measuring the resonance frequency of the NR in the coupled CPB-NR system. Figure 2a illustrates the absorption of the signal current as a function of the detuning *Δ_s _*(*Δ_s _*= *ω_s _*- *ω_q_*). The absorption (Im(*χ*)) has been normalized with its maximum when the control current is resonant with the CPB qubit (*Δ_c _*= 0). Mollow triplet, commonly known in atomic and some artificial two-level system [31,32], appears in the middle part of Figure 2a. However, there are also two sharp peaks located exactly at *Δ_s _*= ±*ω_n _*in the sidebands of the absorption spectrum, which corresponds to the resonant absorption and amplification of the vibrational mode of the NR. Our proposed mass sensing scheme is just based on these new features in the absorption spectrum. An intuitive physical picture explaining these peaks can be given in the energy level diagram shown in Figure 2b. The Hamiltonian of the coupled system without the externally applied current can be diagonalized [33,34] in the eigenbasis of $|\pm ,N_{\pm }\rangle =\;{|\pm \rangle }_{z}⊗e^{\mp (\lambda /\omega_{n})(a^{†}-a)}|N\rangle$, with the eigenenergies *E*_± _= ±*ħ*/2*ω_q _*+ *ħω_n_*(*N *- *λ*_0_), where the CPB qubit states |±〉*_z _*are eigenstates of *σ_z _*with the excited state |+〉*_z _*= |*e*〉 and the ground state |-〉*_z _*= |*g*〉, the resonator states |*N*_±_〉 are position-displaced Fock states. Transitions between |-, *N*_-_〉 and |+, (*N *+ 1)_+_〉 represent signal absorption centered at *ω_c _*+ *ω_n _*(the rightmost solid line in Figure 2b). Besides, transitions between |+, *N*_+_〉 and |-, (*N *+ 1)_-_〉 indicate probe amplification (the leftmost solid line in Figure 2b) because of a three-photon process, involving simultaneous absorption of two control photons and emission of a photon at frequency *ω_c _*- *ω_n_*. The middle dashed lines in Figure 2a corresponds to the transition where the signal frequency is equal to the control frequency. Therefore, Figure 2a provides a method to measure the resonance frequency of the NR. If we first tune the frequency of the control MW current to be resonant with the CPB qubit (*ω_c _*= *ω_q_*) and scan the signal frequency across the CPB qubit frequency, then we can easily obtain the resonance frequency of the NR from the signal absorption spectrum.

**Figure 3:**
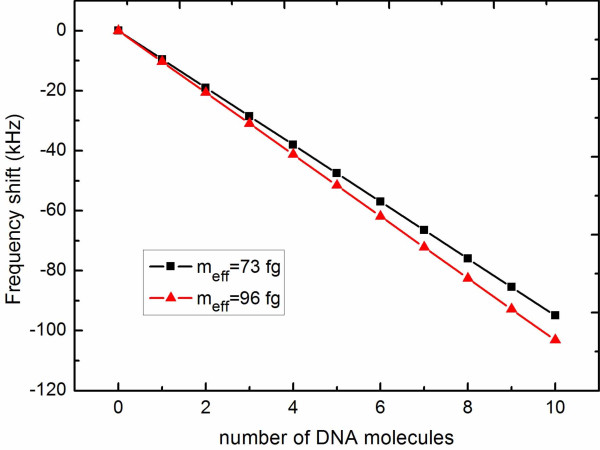
**Plot of frequency shifts versus the number of DNA molecules landing on two different masses of NRs**. Other parameters used are *ω_n _*= 835 MHz, *λ*_0 _= 0.01, *Δ*_c _= 0. *Q *= 5000, *T*_1 _= 0.25 *μ*s, *T*_2 _= 0.05 *μ*s, and *Ω_c _*= 3.

Next, we illustrate how to measure the mass of the particles landing on the NR based on the above discussions. Unlike traditional mass spectrometers, nanomechanical mass sensors do not require the potentially destructive ionization of the test sample, are more sensitive to large biomolecules, such as proteins and DNA, and could eventually be incorporated on a chip [6]. Here, we use the functionalized 1587 bp long dsDNA molecules with mass *m*_DNA _≈ 1659 zg (1 zg = 10^-21 ^g) [35], and assume for simplicity that the mass adds uniformly to the mass of the overall NR and changes the resonance frequency of the NR by an amount given by Equation 19. Figure 2c demonstrates the signal absorption as a function of *Δ_s _*before and after a binding event of ~ 10 functionalized 1587 bp DNA molecules in the vicinity of the resonance frequency of the NR. We can see clearly that there is a resonance frequency shift *Δω *= -95 kHz after the adsorption of the DNA molecules because of the increased mass of the NR. According to Equation 19, we can obtain the mass of the accreted DNA molecule: $\Delta m=-\frac{2m_{\text{eff}}}{\omega_{n}}\Delta \omega =16590\text{zg}$, about the mass of 10 functionalized 1587 bp long dsDNA molecules. Therefore, such a coupled CPB-NR system can be used to weigh the external accreted mass landing on the NR by measuring the frequency shift in the signal absorption spectrum when the control current is resonant with the CPB qubit. Plot of frequency shifts versus the number of DNA molecules landing on two different masses of NRs. Other parameters used are *ω_n _*= 835 MHz, *λ*_0 _= 0.01, *Δ*_c _= 0, *Q *= 5000, *T*_1 _= 0.25 *μ*s, *T*_2 _= 0.05 *μ*s, and *Ω_c _*= 3. Mass responsivity  ℛ is an important parameter to evaluate the performance of a resonator for mass sensing. Figure 3 plots the frequency shifts as a function of the number of DNA molecules landing on the NR for two different kinds of NRs. One is *ω_n _*= 2*π *× 133 MHz (*m*_eff _= 73 fg), the other is *ω_n _*= 2*π *× 190 MHz (*m*_eff _= 96 fg) [2,3]. The mass responsivities, which can be obtained from the slope of the line, are, respectively, |ℛ|≈5.72Hz/zg and |ℛ|≈6.21Hz/zg. Smaller mass of the nanoresonator enables higher mass responsivity. Here, we have assumed that the DNA molecules land evenly on the NR and they remain on it. In fact, the position on the surface of the resonator where the binding takes place is one factor that strongly affects the resonance frequency shift. The maximization in mass responsivity is obtained if the landing takes places at the position where the resonator's vibrational amplitude is maximum. For the doubly clamped NR used in our model, maximum shift is achieved at the center for the fundamental mode of vibration, while the minimum shift exists at the clamping points. This statistical distribution of frequency shifts has been investigated by building the histogram of event probability versus frequency shift for small ensembles of sequential single molecule or single nanoparticle adsorption events [6,7].

**Figure 4:**
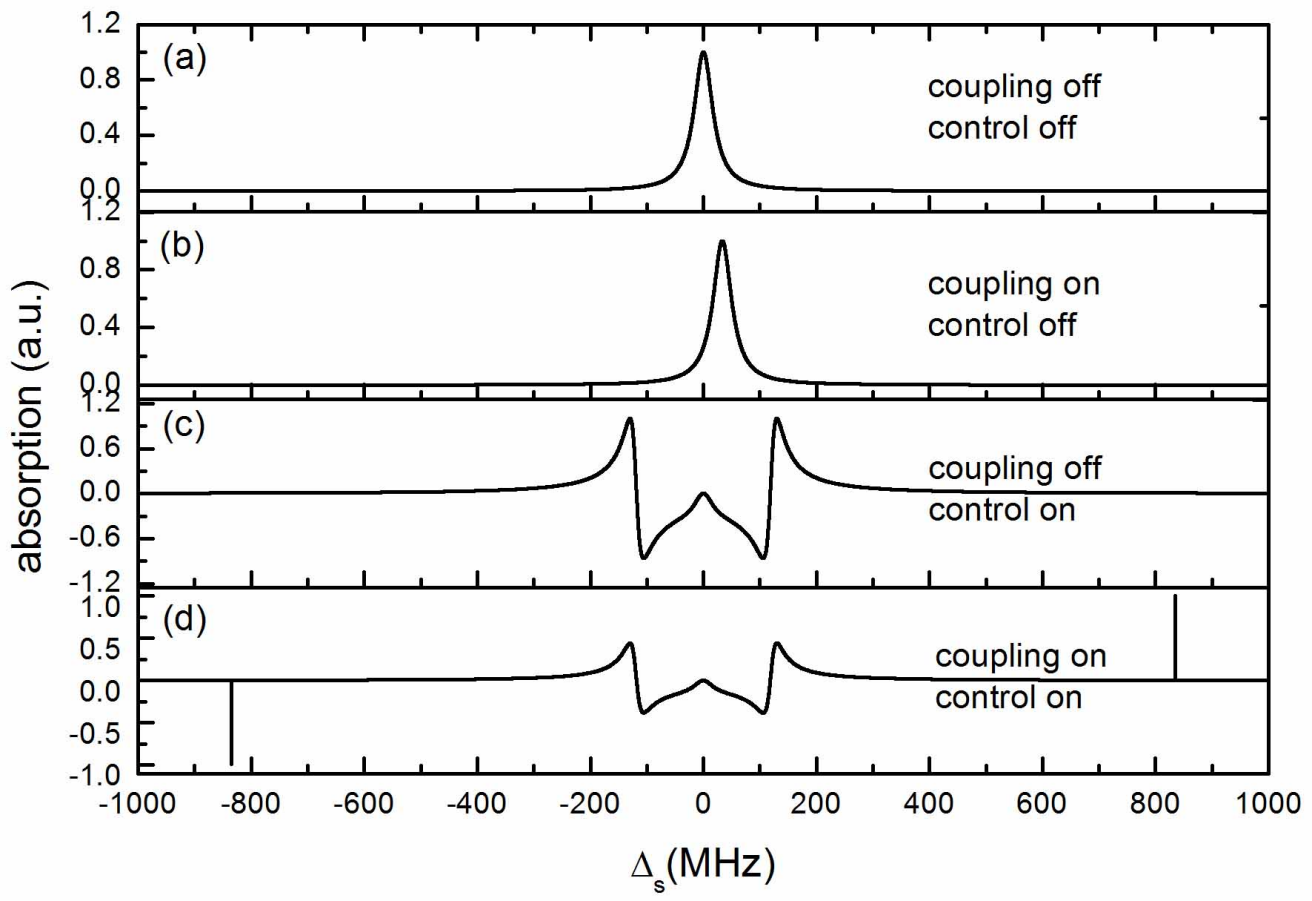
**Signal current absorption spectrum as a function of the detuning ***Δ*_s _**considering the effects of NR and the control field**. Other parameters are *Δ_c _*= 0, *Q *= 5000, *T*_1 _= 0.25 *μ*s, *T*_2 _= 0.05 *μ*s, and *ω_n _*= 835 MHz.

In order to demonstrate the novelty of our proposed mass sensing scheme, we plot Figure 4 to illustrate how the vibration mode of NR and the control current affect the spectral features. Figure 4a shows the absorption spectrum of the signal field through the CPB system without the influence of the NR (coupling off) in the absence of the control field (control off), which shows the standard resonance absorption profile. However, when the coupling turns on, the center of the curve shifts from the resonance *ω*_s _= *ω*_q _a bit, as shown in Figure 4b. This is because of the coupling *λ*_0 _between the CPB and the NR [16,36]. Figure 4c demonstrates the absorption spectrum of the signal field when the control field turns on in the absence of the NR (coupling off). This is the commonly known Mollow triplet, which appears in atomic and some artificial two-level system [31,32]. None of the above situations can be used to measure the resonance frequency of the NR. However, when the coupled CPB-NR system is driven by a strong control field and a weak signal field simultaneously, the resonance frequency of the NR be measured from the absorption spectrum of the signal field, as shown in Figure 4d. The spectral linewidth of the two sideband peaks that corresponds to the resonance frequency of the NR is much narrower than the peak in the center, since the damping rate of the NR is much smaller than the decay rate of the CPB qubit. Therefore, such a coupled CPB-NR system is proposed here to measure the resonance frequency of the NR when the control field is resonant with the CPB qubit (*ω_c _*= *ω_q_*). By measuring the frequency shift of the NR before and after the adsorption of particles landing on it, we can obtain the accreted mass according to Equation 19.

## 4 Conclusion

To conclude, we have demonstrated that the coupled NR-CPB system driven by two MW currents can be employed as a mass sensor. In this coupled system, the CPB serves as an auxiliary system to read out the resonance frequency of the NR. Therefore, the accreted mass landing on the NR can be weighed conveniently by measuring the frequency shifts in the signal absorption spectrum. In addition, the use of on-chip capacitive readout will prove especially advantageous for detection in liquid environments of low or arbitrarily varying optical transparency, as well as for operation at cryogenic temperatures, where maintenance of precise optical component alignment becomes difficult.

## Competing interests

The authors declare that they have no competing interests.

## Authors' contributions

CJ finished the main work of this article, including deducing the formulas, plotting the figures, and drafting the manuscript. BC and JJL participated in the discussion and provided some useful suggestion. KDZ conceived of the idea and participated in the coordination.
